# Use of dietary supplements in Olympic athletes is decreasing: a follow-up study between 2002 and 2009

**DOI:** 10.1186/1550-2783-8-1

**Published:** 2011-02-04

**Authors:** Anni Heikkinen, Antti Alaranta, Ilkka Helenius, Tommi Vasankari

**Affiliations:** 1 The Paavo Nurmi Centre Sports & Exercise Medical Unit, Department of Physiology University of Turku, Kiinamyllynkatu 10, 20520 Turku, Finland; 2 Turku Children's Hospital, Turku University Central Hospital, Kiinamyllynkatu 4-8, 20521 Turku, Finland; 3 The National Institute for Health and Welfare, Mannerheimintie 166, Helsinki, Finland; 4 The UKK Institute for Health Promotion Research, Kaupinpuistonkatu 1, 33500 Tampere, Finland; 5 The Finnish Olympic Committee, Radiokatu 20, 00240 Helsinki, Finland

## Abstract

**Background:**

The aim of this study was to assess the frequency of use of dietary supplements (DS) among large sample of elite Finnish athletes and to describe possible changes in dietary supplement use between the years 2002 and 2009.

**Methods:**

A prospective follow-up study was conducted on Olympic athletes. The first survey was conducted on Olympic athletes in 2002 (N = 446) and the follow-up study was conducted between May 2008 and June 2009 (N = 372).

**Results:**

In 2002, a total of 81% of the athletes used dietary supplements (a mean of 3.37 ± 3.06 DS per user) and in 2009, a total of 73% of the athletes (a mean of 2.60 ± 2.69 per DS user) used them. After adjusting for age-, sex- and sport type, the OR (95% confidence interval, CI) for use of any dietary supplement was significantly less in 2009 as compared with 2002 results (OR, 0.62; 95% CI, 0.43-0.90). Decrease in DS use was observed in all supplement subgroups (vitamins, minerals, nutritional supplements). Athletes in speed and power events and endurance events reported use of any dietary supplement significantly more often than team sport athletes both in 2002 and 2009. In year 2009, the frequency of all dietary supplement use increased when athlete's age increased and the increase was significant in older age groups: of the athletes under 21 years 63%, 21-24 years 83% and over 24 years 90% consumed nutritional supplements.

**Conclusions:**

Based in our study, there seems to be a lowering trend of dietary supplement use among elite Finnish athletes although differences between sport subgroups and age groups are considerable.

## Introduction

Athletes use dietary supplements in order to increase energy, maintain strength, enhance performance, maintain health and immune system and prevent nutritional deficiencies [1-12]. A recent increase in DS use has been observed in various sports and especially among elite athletes [13,6]. There are several studies estimating that supplement use among athletes is common and varies between 59 to 88% multivitamins, minerals, proteins and energy drinks being most common products being consumed [1-12].

Most supplement users consume more than one product [1,4,6,7,9,12,14] and the amount of supplements used varies between age groups, gender and different sports [2-6,10,14,15]. Norwegian study reported a great difference of supplement use between different sport groups: power sport athletes had the most frequent use of supplemental creatine, proteins/amino acids, vitamins and minerals while cross-country skiers had the most frequent intake of iron, vitamin C and fish oils [10].

Athletes are willing to use many kinds of dietary supplements, although researches haven't been able to prove that most supplements perform as claimed. In their recent statement, American dietetic association (ADA) lists ergogenic aids into four groups according to their safety and efficiency: 1. those that perform as claimed; 2. those that may perform as claimed but for which there is insufficient evidence of efficacy at this time; 3. those that do not perform as claimed; and 4. those that are dangerous, banned, or illegal and, therefore, should not be used. Group one contains creatine, caffeine, sport drinks, gels and bars, sodium bicarbonate and proteins and amino acids. On the contrary, group three includes majority of the ergogenic aids currently on the market including widely used ginseng and branched chain amino acids [16]. When it comes to vitamin and mineral supplementation, according to ADA and HC Lukaski using them does not improve performance among individuals who consume nutritionally adequate diets [16,17]. Except for one study [6], no previous follow-up studies exist on trending athletes DS use. In our study, it was interesting to see whether the report concerning purity of dietary supplements [18]made by the International Olympic Committee had an affect on elite Finnish athletes use of DS.

The aim of this study was to assess the frequency of use of dietary supplements among large sample of elite Finnish athletes and to evaluate possible trends in DS use between 2002 and 2009. DS use has not been reported previously in elite Finnish athletes.

## Materials and methods

### Study design for athletes

A prospective follow-up study was conducted in Olympic athletes. The first questionnaire was given for Olympic athletes in 2002 and the follow-up study was conducted between May 2008 and June 2009.

In Finland, the National Olympic Committee supports financially 1) the Finnish national teams of those sport associations which have adequate training organization for athletes to acquire Olympic success in the next Olympic games 2) individual athletes with Olympic medal possibilities but without adequate sport association's training organization 3) future Olympic hopefuls 4) teams with possible success in the Olympic Games. The population of this study comprised all athletes eligible for financial support from the National Olympic Committee. Most athletes completed the questionnaire at their national team camps. If athletes were absent from their national team camps the questionnaire was sent them by mail. Of the athletes, 446 (response rate 90.3%) completed a structured questionnaire in 2002 and 372 (response rate 91.9%) in 2008-2009. Athletes were divided into four groups according to their type of sport. When defining these groups the same classification used previously by our study group was applied: speed and power athletes, endurance athletes, athletes in motor skill demanding events and team sport athletes (Table 1) [19]. The characteristics of the study groups in both study years are given in Table 2. Further description of the inclusion criteria and the study population year 2002 have been described in detail elsewhere [19].

**Table 1:** Participating athletes by types of sport

		Response			Response
Winter Events	N = 126	Rate	Summer Events	N = 246	Rate
Speed and power	Freestyle Speed skating Alpine events	100% (23 of 23)	Speed and power	Judo Track and field (sprinters, hurdles jumpers, throwers, decathletes)	83.2% (89 of 107)
				Wrestling Weight lifting	
				Boxing Taekwondo	
					
Endurance	Biathlon Cross-country skiing Nordic combined	100% (42 of 42)	Endurance	Rowing Badminton Swimming Canoeing Track and field (800 m+)	84.4% (38 of 45)
				Tennis	
					
Motor skills demanding	Figure skating Ski jumping Snow boarding	100% (25 of 25)	Motor skills demanding	Shooting Archery Sailing Fencing	91.7% (44 of 48)
				Horse riding Gymnastics	
Team sports	Ice hockey (women)	94.7% (36 of 38)	Team sports	Volleyball (men) Volleyball (women U-17)	97.4% (75 of 77)
	Ice hockey (men U-20)			Volleyball (men U-17) Handball (women U-17)	
				Hanball (men U-17)	
				Basketball (women U-17)	
				Basketball (men U-17)	

**Table 2:** Characteristics of the study groups

	All athletes		Speed and power events		Endurance events		Motor skills demanding events		Team sport events	
	2002	2009	2002	2009	2002	2009	2002	2009	2002	2009
	N = 446	N = 372	N = 113	N = 112	N = 108	N = 80	N = 73	N = 69	N = 152	N = 111
**Sex (men/women)**	261/185	218/154	82/31	74/38	62/46	45/35	45/28	40/29	72/80	59/52
**Mean (SD) age (yr)**	23(4.5)	21.2 (4.3)	23.8 (4.1)	21.8 (3.7)	23.6 (4.0)	23.5 (4.1)	23.6 (6.5)	21.4 (4.7)	21.6 (3.6)	18.7 (3.7)
**Mean (SD) duration of**	11.7 (4.3)	10.2 (4.5)	12.2 (3.7)	10.8 (4.5)	12.4 (4.6)	11.8 (5.0)	11.9 (5.0)	10.2 (4.2)	10.8 (4.1)	8.2 (3.4)
**active sport career (yr)**										
**Mean (SD) training amount (h-wk ˉ¹)**	15 (6)	14 (5)	15 (4)	14 (4)	17 (5)	16 (4)	15 (7)	14 (5)	14 (6)	13 (6)
**Response rate (%)**	90.3	91.9	89.0	86.2	90.8	92.0	82.0	94.5	95.6	96.5

### Questionnaire

Athletes in our study answered a semi-structured questionnaire, which was based on the Finnish national health survey Health 2000 coordinated by the National Institute for Health and Welfare. The initial questionnaire was tested on national level ice-hockey players and track and field athletes (n = 30) who were not included in the final study. Researcher represented the study to athletes and answered to athlete's questions if clarifications were required. Athletes filled a structured questionnaire after accepting written informed consent. Athletes who received the questionnaire by mail were given the possibility to consult a researcher by phone or e-mail. Athletes filled the questionnaire anonymously. Ethical approval for the study was granted by the ethical committee of University of Turku, Finland.

Questions concerned athlete's dietary supplement use. Athletes were asked to name all vitamins, minerals, nutritional supplements and herbal as well as homeopathic preparations used during previous 12 months.

Dietary supplements were categorized into subgroups for further analysis. The categorization was identical to a Canadian study concerning elite athlete's medication and dietary supplement use in Atlanta and Sydney Olympic games [6]. Dietary supplements were defined as vitamins, minerals and nutritional supplements (including amino acids, proteins, carbohydrates, creatine, caffeine, oils or fatty acids, herbal or homeopathic supplements and other supplements). Supplements that were defined as "herbal supplements" were products mainly derived from plant sources such as echinacea, garlic and ginseng. "Other supplements" included products that couldn't be categorized any other way, such as fibres, beastings and conjugated linoleic acid. "Vitamin supplements" included multivitamins, vitamins A, B, C, D and E, beta-carotenes and antioxidant agents. "Mineral supplements" consisted of iron, calcium, magnesium and other mineral products such as zinc, fluorine, potassium and multi-minerals.

### Statistical methods

Odds ratios (ORs) for use of dietary supplements and their 95% CIs for athlete subgroups in 2009, compared with athlete subgroups in 2002, were analyzed using logistic regression model with the aid of SPSS 16.0 software. Age, sex and type of sport were included in the analysis as independent covariates.

## Results

### Frequency of supplement use in 2002 and 2009

**Figure 1:**
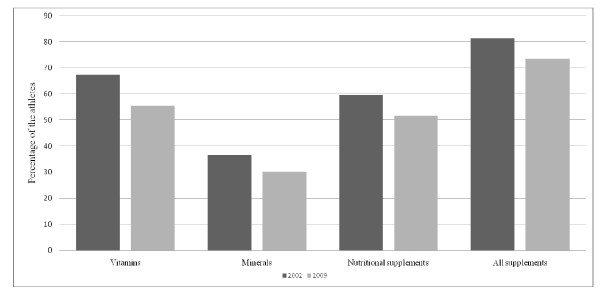
**Dietary supplement use between study years**.

**Figure 2:**
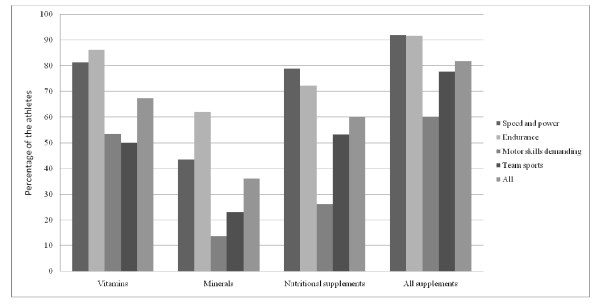
**Dietary supplement use in different sports in 2002**.

**Figure 3:**
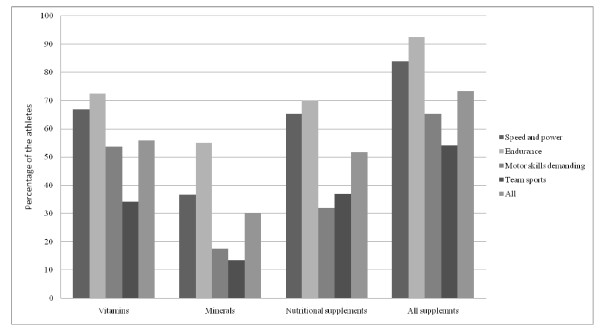
**Dietary supplement use in different sports in 2009**.

The questionnaire was completed by 446 of 494 (90.3%) athletes in 2002 and 372 of 405 (91.7%) athletes in the follow-up study. Of the 446 athletes, 81% reported supplement use during previous 12 months in 2002 and 73% of the 372 athletes in 2009. Decreased consumption of dietary supplements between study years was seen in all subgroups except for amino acids (3.8% in 2002 and 7.3% in 2009), oils and fatty acids (11% and 19%), homeopathic supplements (0.4% and 1.6%), multivitamins (54% and 57%) and antioxidants (0.7% and 2%). Differences in supplement use between study years are illustrated in Figure 1. Dietary supplement use in different sports in 2002 and 2009 are illustrated in Figures 2 and 3.

Mean number of supplements consumed were 3.4 ± 3.1 in 2002 and 2.6 ± 2.7 in 2009. In 2002, the highest amount of different dietary supplements consumed per athlete was 18. In 2009, the highest amount of different dietary supplements was 14. In 2009, among all athletes the most often declared subgroup used was vitamin supplements (56%) and most of the vitamin supplement users consumed multivitamins (57%). Nutritional supplements were used by 52% of the athletes, proteins (38%) and oils and fatty acids (19%) being the biggest subgroups.

### All dietary supplement use

After adjusting for age-, sex- and sport type, the OR (95% CI) for use of any dietary supplement was significantly less in 2009 sample as compared with 2002 sample (OR, 0.62; 95% CI, 0.43-0.90). Athletes in speed and power events and endurance events reported use of any dietary supplement significantly more often than team sport athletes both in 2002 and 2009 (Table 3). In 2002, all DS use among athletes in skill-based sports was significantly less than among athletes in team sports (OR, 0.46; CI 0.25-0.85). Neither in 2002 nor 2009 was any significant difference observed between females and males in DS use. In 2002 sample group, there was no significant difference in any dietary supplement use between age groups (Table 3). However, in 2009 sample group, athletes over 24 years consumed significantly more dietary supplements than athletes in under 21 years.

**Table 3:** Logistic regression model on DS use

	Vitamins		Minerals		Nutritional supplements		All dietary supplements	
**Characteristic**	**OR**	**95% CI**	**OR**	**95% CI**	**OR**	**95% CI**	**OR**	**95% CI**
***Sex***								
**Men (2002)**	1		1		1		1	
**Men (2009)**	1		1		1		1	
**Women (2002)**	1.32	0.85-2.06	2.13	1.36-3.33	0.54	0.35-0.83	0.92	0.55-1.55
**Women (2009)**	2.30	1.42-3.72	2.24	1.36-3.68	0.58	0.37-0.91	1.21	0.72-2.02
***Age (yr)***								
**Under 21 (2002)**	1		1		1		1	
**Under 21 (2009)**	1		1		1		1	
**21-24 (2002)**	1.28	0.76-2.16	1.54	0.91-2.62	1.34	0.80-2.23	1.19	0.63-2.27
**21-24 (2009)**	1.66	0.95-2.90	1.16	0.63-2.14	2.47	1.40-4.34	1.90	0.97-3.70
**Over 24 (2002)**	0.86	0.51-1.46	1.63	0.95-2.80	0.92	0.55-1.54	0.70	0.38-1.30
**Over 24 (2009)**	6.77	3.22-14.23	2.15	1.14-4.07	4.43	2.31-8.50	3.18	1.38-7.33
***Type of sport***								
**Team Sport (2002)**	1		1		1		1	
**Team Sport (2009)**	1		1		1		1	
**Speed and power (2002)**	4.67	2.56-8.52	3.85	1.90-7.82	2.76	1.55-4.91	3.37	1.50-7.57
**Speed and power (2009)**	3.71	2.02-6.81	2.83	1.60-5.03	2.25	1.25-4.05	3.65	1.89-7.03
**Endurance (2002)**	6.50	3.40-12.42	6.56	3.03-14.2	2.15	1.25-3.72	3.30	1.48-7.32
**Endurance (2009)**	3.13	1.54-6.36	5.98	3.38-10.58	2.11	1.06-4.20	6.73	2.60-17.48
**Skill-based (2002)**	1.26	0.71-2.22	1.25	0.53-2.94	0.29	0.16-0.55	0.46	0.25-0.85

### Vitamin use

After adjusting for age-, sex- and sport type, the OR (95% CI) for vitamin use was significantly less in 2009 sample group as compared with 2002 sample (OR, 0.62; 95% CI, 0.45-0.85). Both in 2002 and 2009, vitamin use was significantly more frequent among speed and power athletes and endurance athletes as compared with team sport athletes (Table 3). Vitamin use was more frequent among female athletes than male athletes in 2009 (OR 2.30; 95% CI 1.42-3.71). In 2009, athletes in age group over 24 years took significantly more vitamins than athletes in age group under 21 years (OR 6.77; 95% CI 3.22-14.23). In 2002, no significant difference was seen in vitamin use between different age groups.

### Mineral use

There was a trend for less use of minerals in 2009 as compared with 2002 sample group (adjusted OR, 0.77; 95% CI, 0.56-1.08). Mineral use was significantly more frequent among speed and power athletes and endurance athletes when compared against team sport athletes, both in 2002 and 2009 (Table 3). Women used significantly more often minerals than men in 2002 (OR, 2.13; 95% CI, 1.36-3.33) and 2009 (OR, 2.24; 95% CI, 1.36-3.68). In 2009, athletes over 24 years used minerals significantly more often than athletes in the youngest age group.

### Nutritional supplement use

No significant difference was found in athlete's nutritional supplement use in the age-, sex- and sport type adjusted OR (95% CI) when 2009 sample group was compared with 2002 sample group (OR, 0.77; 95% CI, 0.56-1.04). Speed and power athletes as well as endurance athletes consumed significantly more often nutritional supplements than team sport athletes in both in 2002 and 2009 (Table 3). Women took significantly less nutritional supplements than men both in 2002 and 2009 (2002, OR, 0.54; 95% CI, 0.35-0.83 and 2009 OR, 0.58; 95% CI, 0.37-0.91). Nutritional supplement use was significantly more frequent among athletes in age groups 21-24 years and over 24 years in 2009 when compared with athletes in age group under 21 years. In 2002, no significant difference in nutritional supplement use between age groups was seen.

## Discussion

The main finding in our study was the decreased supplementation among elite Finnish athletes. Significant decrease was observed in all supplement use (81% in 2002 and 73% in 2009) and vitamin use (67% in 2002 and 55% in 2009). The decrease in DS use may be partly explained with athlete's increased awareness concerning purity issues and contamination of dietary supplements [18]. Between study years, there were no policy changes made by the Finnish Olympic Committee concerning athlete's DS use.

When comparing our results with a study that reported Canadian Olympic athlete's dietary supplement use in Atlanta (69%) and Sydney Olympic games (74%), it can be seen that rates of supplement use among elite Finnish athletes are still high [6]. We found no other follow-up studies comparing trends in elite athlete's DS use. In our survey, nutritional supplement use was significantly higher among males than females both in 2002 and 2009 whereas the Canadian study reported all DS use being slightly more common among female athletes both in Atlanta and Sydney Olympic games.

To our knowledge, our study is one of the first to compare a large number of elite athletes and their supplement use between different sport groups and different time periods. When comparing the amount of study population in our study with other surveys concerning elite athlete's supplement use, it was seen that there are only two studies that had larger study population that we had [4,15]. Because the response rates were high in both study years, the conclusions can be applied to the entire group of elite Finnish athletes.

The characteristics of participants of our study were similar to other studies of with elite athletes [1,4-6,9,10,20]. In 2002, there was a mean of 3.4 DS per athlete, whereas in 2009 the mean amount was decreased to 2.6 DS per athlete. The maximum amount of different DS consumed by an individual athlete decreased as well. In our initial survey one athlete consumed 18 different DS, whereas in follow-up study one athlete consumed 14 different products.

Most frequent vitamin and mineral as well as overall dietary supplement users in both study years were endurance athletes and speed and power athletes. Similarly to Huang et al's report [6], it seems that athletes competing in sports that involve endurance-type of activity and that can be classified as single sports are more likely to use dietary supplements. This is also supported with the fact that in our study team sport athletes consumed less DS. However, it was interesting to find that between study-years athletes in motor skills demanding sports increased their frequency of supplement use. This may be an evidence of a spreading culture of supplement use as athletes who have not traditionally used supplement start adding supplements into their diet.

Most often reported products by our study population during both study years were multivitamins (54% in 2002 and 57% in 2009), proteins (47% and 38%) and vitamin C (28% and 24%). These findings are in line with literature except for carbohydrates which were reported infrequently by our study participants [1-7,10-12,15]. It may be assumed that there was an underreporting in athletes' carbohydrate use since many of the athletes may not consider high levels of carbohydrates containing sport drinks as nutritional supplements. This is supported with the fact that an American study made in 2004 with college athletes reported that 33% of the athletes didn't consider fluid and caloric replacement products (such as Energy mix, Gatorade, Recovery mix) as dietary supplements [5].

One of the findings in our study was the effect of athlete's age in DS consumption rate. In 2002, there was no statistical difference between age groups when examining the frequency of dietary supplementation. In 2009, the consumption of DSs increased significantly in older age groups. Similarly, a Canadian study made in 2007 with high performance elite athletes and a German study made in 2009 with young elite athletes as well as a recent international study made with track and field athletes reported higher rate of DS use among older athletes than with younger athletes [1,4,14]. A study with young elite athletes between ages 12-21 reported 48.1% using at least one supplement [9]. Similarly, a study made with adolescent athletes in central Nebraska reported only 27% of the athletes having used supplements in the past [21]. These rates of supplementation are considerably lower than percentages of supplementation made with older athletes [4,6,8,10,11,15]. In our study, it was also found that in 2002 athletes in age group of 21-24 years were most frequent DS users, whereas in 2009 athletes in the oldest age group (over 24 years) were more likely to use supplements. Because elite athletes took part in our study in both study years, part of the result may be explained with the fact that athletes who were in age group of 21-24 years in 2002 were in the oldest age group when the research was made again in 2009.

For more than a decade it has been known that nutritional supplements (NS) can also contain doping substances. Because of the possible side-effects and non-intentional positive doping results this topic has been widely discussed and various studies have pointed out the seriousness of the problem [17,22-24]. Geyer et al. reported the results of wide international study sponsored by International Olympic Committee concerning the purity of non-hormonal nutritional supplements. Of the 634 samples analyzed 14. 8% contained prohormones not declared on the label. Most of the contaminated supplements (68.1%) contained prohormones of testosterone and contamination was found in all kinds of NS [18]. Baume et al. found similar results in their studies as three of 103 dietary supplements screened contained metandienone and 18 of the products contained precursors or metabolites of testosterone or nandrolone [22]. Although the amounts of the prohormones in NS are mostly low, the excretion studies have shown that the amount of their urine metabolites can rise high because of the high recommended dosages of the NS which lead to positive doping results [18,22].

In their recent paper, Petroczi et al pointed out the lack of surveillance on the dietary supplement market and established the complicated legislation concerning food supplements in European Union [24]. As DS use among Finnish elite athletes seems to be remarkably high, the risk of contaminated supplements must be taken seriously and attention must be taken to athlete's supplement use and dietary education.

### Limitations of the study

When collecting data for the follow-up study our main intention was to keep the source population similar with the study population in 2002. However, between study years the National Olympic Committee had somewhat elevated the criteria for financial support and therefore, fewer small sport federations received support than previously. This is why the study population slightly decreased in follow-up study. However, subgroup sizes between study years (speed and power athletes, endurance athletes, athletes in motor skill demanding events and team sport athletes) were quite comparable. In addition, the study populations in both study years were high enough to explain differences of 5% or less between groups.

There were differences in athlete's ages: mean age of all athletes was lower in follow-up study (23.0 vs. 21.2 years) (Table 2) the difference was greatest in team sport athletes (21.6 vs.18.7 years). Since rates of DS use were significantly lower among younger than older athletes, decreased total DS use between study years may partly be explained by the fact that there were younger athletes in the follow-up study. Lower mean age of the athletes may also explain lower mean training hours per week and shorter duration of active sport career of the athletes in 2009 (Table 2). However, it should be noted that all statistical analyses carried out was done with adjusting for age.

In our survey, athletes were asked to name all dietary supplements, all vitamins, minerals and herbal and homeopathic preparations used during previous 12 months without examples given. In other studies with elite athletes, there are surveys that gave examples or listed supplements they wanted athletes to name in their questionnaire [1,5,12,21]. A comparison with these studies, the absence of examples may have caused some underreporting of supplement use.

## Conclusion

Our study presents the results of follow-up study made with a large sample of elite athletes representing various different sports. According to these results, dietary supplementation among elite athletes seems to be diminishing, especially in younger age groups, but the frequency of supplement use varies between different sport groups being highest among endurance athletes and lowest among team sport athletes. In Finland, male athletes use more nutritional supplements whereas female athletes use more vitamins and minerals.

Compared with other studies with elite athletes, the percentage of dietary supplements used among Finnish Olympic athletes is high. Since the purity of nutritional supplements cannot be guaranteed, professional nutritional counseling is needed to avoid irrational and potentially unsafe practices of dietary supplement use. Further investigations are needed for evaluating elite athlete's dietary supplement use. Sport nutritionist involvement is required to ensure well balanced diet for high training athletes.

## Competing interests

The authors declare that they have no competing interests.

## Authors' contributions

All authors contributed the study design and AA, AH and TV were responsible for the data collection. AH and AA were responsible for the statistical analysis. All authors reviewed and contributed to the final manuscript. All authors have read and approved the final manuscript.

## References

[B1] Braun H Koehler K Geyer H Kleiner J Mester J Schanzer W Dietary Supplement use among Elite Young German Athletes Int J Sport Nutr Exerc Metab 2009 19 97 109 19403956 10.1123/ijsnem.19.1.97

[B2] Dascombe BJ Karunaratna M Cartoon J Fergie B Goodman C Nutritional Supplementation Habits and Perceptions of Elite Athletes within a State-Based Sporting Institute J Sci Med Sport 2010 13 274 80 10.1016/j.jsams.2009.03.005 19775936

[B3] Duellman MC Lukaszuk JM Prawitz AD Brandenburg JP Protein Supplement Users among High School Athletes have Misconceptions about Effectiveness J Strength Cond Res 2008 22 1124 1129 10.1519/JSC.0b013e31817394b9 18545198

[B4] Erdman KA Fung TS Doyle-Baker PK Verhoef MJ Reimer RA Dietary Supplementation of High-Performance Canadian Athletes by Age and Gender Clin J Sport Med 2007 17 458 464 10.1097/JSM.0b013e31815aed33 17993788

[B5] Froiland K Koszewski W Hingst J Kopecky L Nutritional Supplement use among College Athletes and their Sources of Information Int J Sport Nutr Exerc Metab 2004 14 104 120 15129934 10.1123/ijsnem.14.1.104

[B6] Huang SH Johnson K Pipe AL The use of Dietary Supplements and Medications by Canadian Athletes at the Atlanta and Sydney Olympic Games Clin J Sport Med 2006 16 27 33 10.1097/01.jsm.0000194766.35443.9c 16377972

[B7] Nieper A Nutritional Supplement Practices in UK Junior National Track and Field Athletes Br J Sports Med 2005 39 645 649 10.1136/bjsm.2004.015842 16118303 PMC1725305

[B8] Petroczi A Naughton DP Mazanov J Holloway A Bingham J Performance Enhancement with Supplements: Incongruence between Rationale and Practice J Int Soc Sports Nutr 2007 4 19 10.1186/1550-2783-4-19 17997853 PMC2214727

[B9] Petroczi A Naughton DP Pearce G Bailey R Bloodworth A McNamee M Nutritional Supplement use by Elite Young UK Athletes: Fallacies of Advice regarding Efficacy J Int Soc Sports Nutr 2008 5 22 10.1186/1550-2783-5-22 19077317 PMC2654424

[B10] Ronsen O Sundgot-Borgen J Maehlum S Supplement use and Nutritional Habits in Norwegian Elite Athletes Scand J Med Sci Sports 1999 9 28 35 10.1111/j.1600-0838.1999.tb00203.x 9974194

[B11] Striegel H Simon P Wurster C Niess AM Ulrich R The use of Nutritional Supplements among Master Athletes Int J Sports Med 2006 27 236 241 10.1055/s-2005-865648 16541381

[B12] Tian HH Ong WS Tan CL Nutritional Supplement use among University Athletes in Singapore Singapore Med J 2009 50 165 172 19296032

[B13] Berglund B Sports Medicine Update Scand J Med Sci Sports 2001 11 369 371 10.1034/j.1600-0838.2001.110609.x 11782270

[B14] Tscholl P Alonso JM Dollé G Junge A Dvorak J The use of drugs and nutritional supplements in top-level track and field athletes Am J Sports Med 2010 38 133 140 10.1177/0363546509344071 19812387

[B15] Petroczi A Naughton DP The Age-Gender-Status Profile of High Performing Athletes in the UK Taking Nutritional Supplements: Lessons for the Future J Int Soc Sports Nutr 2008 5 2 10.1186/1550-2783-5-2 18186936 PMC2263015

[B16] American Dietetic Association, Dietitians of Canada, American College of Sports Medicine Rodriguez NR Di Marco NM Langley S American College of Sports Medicine Position Stand. Nutrition and Athletic Performance Med Sci Sports Exerc 2009 41 709 731 10.1249/MSS.0b013e31890eb86 19225360

[B17] Lukaski HC Vitamin and Mineral Status: Effects on Physical Performance Nutrition 2004 20 632 644 10.1016/j.nut.2004.04.001 15212745

[B18] Geyer H Parr MK Mareck U Reinhart U Schrader Y Schanzer W Analysis of Non-Hormonal Nutritional Supplements for Anabolic-Androgenic Steroids - Results of an International Study Int J Sports Med 2004 25 124 129 10.1055/s-2004-819955 14986195

[B19] Alaranta A Alaranta H Palmu P Alha P Pietila K Heliovaara M Helenius I Asthma Medication in Finnish Olympic Athletes: No Signs of Inhaled beta2-Agonist Overuse Med Sci Sports Exerc 2004 36 919 924 10.1249/01.MSS.0000128250.17793.47 15179158

[B20] Tsitsimpikou C Tsiokanos A Tsarouhas K Schamasch P Fitch KD Valasiadis D Jamurtas A Medication use by Athletes at the Athens 2004 Summer Olympic Games Clin J Sport Med 2009 19 33 38 10.1097/JSM.0b013e31818f169e 19124981

[B21] Scofield DE Unruh S Dietary Supplement use among Adolescent Athletes in Central Nebraska and their Sources of Information J Strength Cond Res 2006 20 452 455 16686580 10.1519/R-16984.1

[B22] Baume N Mahler N Kamber M Mangin P Saugy M Research of Stimulants and Anabolic Steroids in Dietary Supplements Scand J Med Sci Sports 2006 16 41 48 10.1111/j.1600-0838.2005.00442.x 16430680

[B23] de Hon O Coumans B The Continuing Story of Nutritional Supplements and Doping Infractions Br J Sports Med 2007 41 800 805 10.1136/bjsm.2007.037226 17957017 PMC2465258

[B24] Petroczi A Taylor G Naughton DP Mission impossible? Regulatory and enforcement issues to ensure safety of dietary supplements Food Chem Toxicol 2010 in press 10.1016/j.fct.2010.11.014 21087651

